# A Deep Learning-Based Fault Detection Model for Optimization of Shipping Operations and Enhancement of Maritime Safety

**DOI:** 10.3390/s21165658

**Published:** 2021-08-23

**Authors:** Panayiotis Theodoropoulos, Christos C. Spandonidis, Fotis Giannopoulos, Spilios Fassois

**Affiliations:** 1 Prisma Electronics SA, Leof. Poseidonos 42, 17675 Kallithea, Greece; rdprojects@prismael.com (P.T.); fotis.giannopoulos@prismael.com (F.G.); 2 Department of Mechanical Engineering and Aeronautic, University of Patras, 26504 Patras, Greece; fassois@upatras.gr

**Keywords:** deep learning, condition monitoring, maritime, Convolutional Neural Network (CNN), fault detection

## Abstract

The ability to exploit data for obtaining useful and actionable information and for providing insights is an essential element for continuous process improvements. Recognizing the value of data as an asset, marine engineering puts data considerations at the core of system design. Used wisely, data can help the shipping sector to achieve operating cost savings and efficiency increase, higher safety, wellness of crew rates, and enhanced environmental protection and security of assets. The main goal of this study is to develop a methodology able to harmonize data collected from various sensors onboard and to implement a scalable and responsible artificial intelligence framework, to recognize patterns that indicate early signs of defective behavior in the operational state of the vessel. Specifically, the methodology examined in the present study is based on a 1D Convolutional Neural Network (CNN) being fed time series directly from the available dataset. For this endeavor, the dataset undergoes a preprocessing procedure. Aspiring to determine the effect of the parameters composing the networks and the values that ensure the best performance, a parametric inquiry is presented, determining the impact of the input period and the degree of degradation that our models identify adequately. The results provide an insightful picture of the applicability of 1D-CNN models in performing condition monitoring in ships, which is not thoroughly examined in the maritime sector for condition monitoring. The data modeling along with the development of the neural networks was undertaken with the Python programming language.

## 1. Introduction

The maritime industry is one of the most crucial pillars of modern economy. To substantiate the level of impact, in 2015, 80% of merchandise trade [1] and 90% of the European Union’s (EU) external trade were transported by ships, illustrating the importance of the marine sector for both the European and global markets [2]. An essential requirement of the maritime industry is the reliability and availability of vessels. Safety-oriented and environmental regulations have become more stringent during recent years, demanding increasingly higher regulation for the condition and operation of ships to achieve higher safety standards. As a result, efficient, precise, and timely maintenance is becoming of paramount importance [3]. One of the most critical problems in the maritime industry is the adoption of the maintenance schedule. 

Initially, corrective maintenance policies were adopted, in which maintenance was performed after the occurrence of breakdowns. Inevitably, this approach incurs high costs, downtime, and in some cases, hazardous operations. A more sophisticated approach, and currently the most common, is Preventive Maintenance (PM). In PM, a component is replaced when it is considered to have reached the end of its useful lifetime. The most widely adopted and conservative technique is to estimate the mean time to failure of a component according to experience with components of the same type. Despite a safer approach, it can result in unnecessary costs as well, particularly if a very conservative estimation is used. Moreover, this method does not guarantee a decrease in the number of breakdowns in a fleet, as the replacement of equipment can still be performed too late. Naturally, this method is based on a tradeoff between the number of failures and the lifetime estimation of the components on board. However, a favorable balance between the two is not easy to achieve, mainly due to the different operating conditions between any two vessels. For the maritime domain, a rather new approach is Condition Based Maintenance (CBM), where the actual condition of an asset (vessel) is monitored in real-time to decide the level of required maintenance [4]. While being able to achieve significant maintenance cost reductions, CBM has strict requirements, such as the use of a multisensor monitoring approach for each component onboard, due to the multiple failure modes that are manifested using different monitoring technologies [5]. As such, successful adoption of CBM is strongly connected with the use of sophisticated models that can diagnose the health status of a component based on data from sensor networks onboard. Following rapid development progress, it has been proved that Artificial Intelligence (AI) and machine learning models have numerous applications in all engineering fields [6,7,8,9,10,11], producing remarkable results. More specifically, one prominent field in which AI-based solutions have been producing reliable and accurate results, surpassing other methodologies, is the failure detection and classification domain [12,13,14,15]. Following this trend, the maritime industry is evaluating different AI-based solutions for improving the quality of their operations both in terms of performance efficiency and safety assurance [16]. Considering the wide variety of systems on-board, Machine Learning (ML) approaches that apply to the maritime sector are practically endless. Data-driven techniques and ML-related research in marine engineering are mainly featured on subjects such as energy and performance optimization of the vessel [17], albeit there are studies in which emphasis has been given on solutions regarding the task of fault detection approaches [18,19]. Several studies have demonstrated the successful employment of ML-based approaches in maritime maintenance, to improve the maintenance strategy of marine diesel engines [20], hull condition assessment [21], or the combination of the propeller, hull, gas turbine, and gas turbine compressor [5,22]. The use of ML for fault detection and isolation on gas turbines (prevalent in naval applications) has been extensively studied [23]; rolling element bearings are among the most well-studied components in the literature [24], asynchronous [25], and synchronous [26] motors, and batteries [27], have seen successful applications of ML. ML applications will play a key role in the maritime sector during the coming decades for the estimation and degradation modeling of assets [28,29]. 

**Figure 1:**
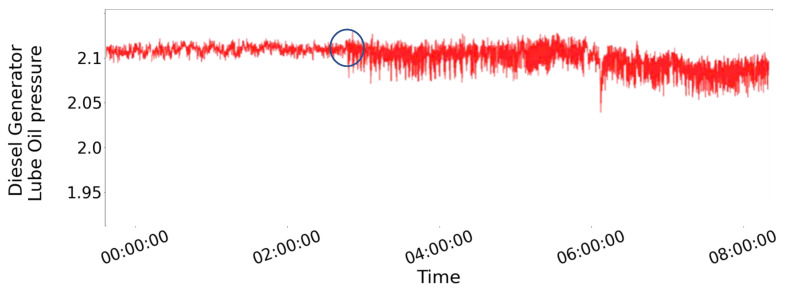
Demonstration of a trigger event from an actual signal collected from the D/G LO pressure parameter onboard a bulk carrier.

Working toward the same direction, in our previous work we investigated how different Deep-Learning (DL) models could provide enhanced information for the support of decision-making [30] within the maritime industry, based on deep learning algorithms. It was shown that the method could accurately predict issues that are caused by long-term degradation (e.g., hull performance) but its effectiveness when instantaneous faults occurred was limited. In this study, our primary focus is to transcend our previous work and progress one step further in the preventive maintenance spectrum implementing condition-based monitoring aspiring to detect failure proneness. It is aspired to develop a CBM scheme to monitor the state of the vessel under study and identify suspicious patterns that indicate failure proneness. More specifically, the signals of the temperature of the main engine’s cylinders are monitored, trying to identify patterns in the signals that signify proneness to failure of the monitored part of the machinery. The type of degradation aspired to detect is either caused by trigger events, such as impacts, or by fracture propagation. These types of degradation are demonstrated in the monitored signal by the inflated jittering in the signal. Figure 1 demonstrates the type of deficiency we aspire to detect with our methodology, illustrating the occurrence of a trigger, even to the actual signal collected from the Diesel Generator (D/G) Lube Oil (LO) pressure over approximately 2 days, as the impairment manifests itself through the inflated volatility due to a crack in the crankshaft. The blue circle in the figure shows the commencement of damage. According to the vessel operator, the fault was not visible by the instruments onboard and the root cause was found only after costly unscheduled maintenance.

**Figure 2:**
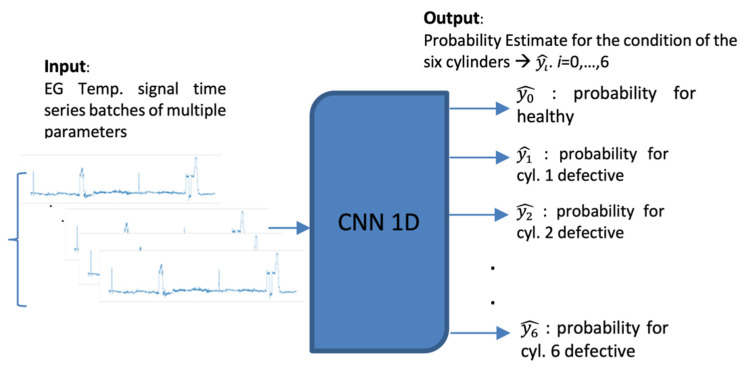
Overview of the 1D-CNN proposed in this study.

The proposed implementation employs a unidimensional (1D) Convolutional Neural Network (CNN) that monitors of the condition of the main engine, which inspects the signal of the temperature of the main engine’s cylinders (Figure 2). This CBM system facilitates the implementation of a short-term approach, as the time series can be divided into smaller batches without affecting adversely the performance of the model. In this instance, we attempt to classify the signal obtained from the exhaust gas temperature of the six cylinders of the ship’s main engine, detecting divergence from the healthy state, caused by degradation and wear, assuming that in the context of our study case, the cases examined refer to occasions with only up to one cylinder being degraded. This form of deficiency is typically expressed as increased jitter of the monitored signal. The networks receive as inputs time series batches of the six signals of the exhaust gas temperature of the six cylinders representing the same time interval. The output is the probabilities of the validity for each possible state of the seven distinct states. Let the available label range be 0–6, then the label 0 signifies a well-functioning vessel, or equivalently, the label i expresses that the respective cylinder i is defective (i.e., 1–6). Therefore, it is aimed to not only identify the operational condition of the ship through the classification of time series batches derived from the dataset, but also, in case of deficiency, to explicitly localize the damage. In this regard, the prominence of CNNs in pattern recognition is examined through their capacity to extract and represent features at high levels of abstraction. To the knowledge of the authors, no similar effort has been presented in the maritime industry. The remainder of the paper is structured as follows: In Section 2, the theoretical background of the model is provided along with a description of the evaluation metrics adopted. In Section 3, a presentation of the data collection and preprocessing procedures are provided. Both steps are required in almost every data-driven analysis, assuring optimal quality of the data utilized. In Section 4, a descriptive overview of the function of CNNs is provided followed by the results yielded in the parametric inquiry. Benchmarking with different optimization models is also provided in this section. Concluding remarks and future steps are discussed in Section 5. 

## 2. Theoretical Background of Convolutional Neural Networks

### 2.1. Model Overview

**Figure 3:**
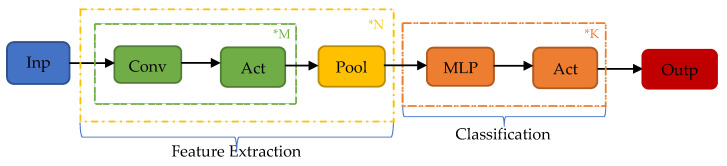
Conceptualization of a CNN.

CNNs are a class of deep learning neural networks that display great capabilities in the task of pattern recognition. They are analogous to traditional Artificial Neural Networks (ANN), in the sense that they are composed of neurons learning in a self-optimizing fashion, updating the weights assigned to these neurons through a back-propagation algorithm, trying to emulate biological processes. Their most significant trait is unequivocally their ability to extract regional features, enabling researchers and developers to deal with complex tasks that are not possible with classic ANNs. Figure 3 illustrates the conceptualization of a CNN and its operations.

As shown, having inserted the input into the neural network, the feature extraction-related elements of the architecture are actuated. First, convolutional layers undertake the task of feature regional recognition. Subsequently, the output of the convolutional layers is received by the activation functions that introduce nonlinearity in the feature learning task. This sequence can be repeated *M* times before the pooling layers receive the output of the final activation function, which reduces the dimensionality of the tensor that propagates through the network, attempting to decrease the computational cost. In turn, the process of *Convolution–Activation–Pooling* is plausibly repeated *N* times before this pattern of alternating convolutional and pooling layers is concluded with the output of the last set of layers flattened into a 1D array vector, to be passed into a fully connected dense multilayer perceptron consisted of *K* layers, executing the classification. The operations of these procedures are described with greater detail in the following paragraphs.

Input: While the great benefits of CNN utilization are well known when processing 2D data, such as images, it has been shown in industrial and academic applications that a modified version of CNN can handle 1D data as well (e.g., in the classification of financial time series or natural language processing). 

Feature extraction: As the name of the network suggests, the convolution layer is a fundamental component of a CNN, with multiple linear and nonlinear operations taking place, contributing to the feature extraction. First, the convolution is characterized as a linear element-wise multiplication, with a small array called the kernel, which glides through the input tensor of the layer and convolves the value of each time step of the time series. The elements of the array, which occurred from the element-wise multiplication of the portion of the input tensor with the weights of the kernel, are summed to obtain the value of the output tensor in the corresponding position. Convolution on a time series is taking place as follows:

(1) $$F\left(x\right)=K{}^{\circ}S\left(x\right)=\overset{N}{\underset{i=-N}{\sum }}K\left(i\right)S\left(x-i\right)\;$$

The output tensor is called a feature map. Subsequently, the output of the convolution operation is fed into a nonlinear activation function. For many years, sigmoid and tanh were mainly chosen as they emulate the behavior of a biological neuron. However, problems caused in the back-propagation regarding the gradient diminishing when the architecture of the network is deep, prompted researchers to adopt a different activation function to introduce nonlinearity. Recently, the rectifier linear unit (ReLU) has started to gain popularity.

Typically, in CNN models, pooling layers alternate with convolutional layers. Pooling layers undertake to downsample, reducing in-plane dimensionality of feature maps, thus diminishing the complexity for the forthcoming layers. The pooling procedure renders the model’s translational invariant, and also offers immunity to small shifts and distortions. There are several types of pooling, the most popular form is max pooling. A window functioning similarly to the kernel mentioned in the convolutional layer glides through the feature map and keeps the maximum value from each patch, whereas all other elements are discarded.

Classification: After this pattern of alternating convolutional and pooling layers is concluded, the output feature map of the last set of layers is flattened into a 1D array vector, which serves as the input of a traditional Feed-Forward Neural Network (FFNN) that is used to execute the classifications. 

The last notable part of the information in the forward propagation journey is the loss function. The main objective of the optimization procedure is the minimization of the loss function. The Categorical Cross-Entropy (CCE) loss function is introduced to better conceptualize and comprehend how the proximity between predicted and real values is quantified. The selection of the aforementioned loss function was made due to the nature of our application, which is a multilabel classification problem. Assuming that the total number of classes is M and the observations contained in the dataset are N, then accept that the vector containing the target values from the dataset is denoted as $y=[y_{1},\;y_{2},$ …, $y_{n}]$. Plausibly, the predicted values of the network are the output values of the last layer. Assuming that the network has L layers, and following the notation previously used, we express the predicted values as $a^{\left[L\right]}=\left[a_{1}^{\left[L\right]},\;a_{2}^{\left[L\right]},\;\ldots ,\;a_{n}^{\left[L\right]}\right]$. Accepting CCE as our loss function then gives:

(2) $$E=CCE\left(y,a^{\left[L\right]}\right)=–\overset{M}{\underset{i=1}{\sum }}y_{i}\log\left(a_{i}^{\left[L\right]}\right)$$

Training a network is a process in which the model is self-improving. After several epochs, the output predictions and ground-truth labels should converge, hence minimizing the loss function. After the conclusion of the forward pass, the backpropagation takes place, where through the calculation of the gradient of the loss between the output estimations of the model, the model updates its learnable parameters, namely weights and biases, attempting to minimize loss by implementing gradient descent, which is used to update the learnable parameters of the model to minimize the loss, hence achieving better accuracy. Mathematically, a gradient is a partial derivative; we calculate the partial derivative of the loss function concerning each of the learnable parameters, and then the respective learnable parameter is updated with an arbitrary step size dictated by the learning rate, which is a hyperparameter:

(3) $$p=p-a\;\frac{\partial L}{\partial p}\;$$

where *p* represents any learnable parameter and α denotes the learning rate of the optimizer of the model. In Appendix A, Table A1 presents a comprehensive overview of the operations and the learning procedure of a 1D-CNN, demonstrating both the forward and back-propagation components of the network.

### 2.2. CNN Evaluation Metrics

Different metrics have been used to evaluate and assess the models after the completion of the tuning procedure in the framework of this study. Receiver Operating Characteristic curves (ROC) along with the respective Area Under Curve (AUC) values are employed as they facilitate comparisons among multiple models. Since the models provide not only the condition of the vessel, but in the case of deficiency the location of the defective cylinder as well, this problem is considered as a multilabel classification task. However, the creation of the ROC curves required a reduction to binary classification regarding whether the network managed to assign the correct label. 

Furthermore, the introduction of a diverse set of metrics is recommended, as this practice provides a more thorough understanding of our models’ performance, avoiding the pitfall of being misled in the case of consulting only one metric. The basis of all the implemented metrics is the confusion matrix, used to describe the performance of a classifier. The confusion matrix of a multiclass classification task with *n* output classes is defined as an n × n array that tracks the correct evaluation of the model and the misclassifications.

The abbreviations TP, TN, FP, and FN mean True Positive, True Negative, False Positive, and False Negative, respectively. What we must do is find TP, TN, FP, and FN for each class. More specifically, concerning each class the TP quantity refers to the observations where the model successfully identified the true label; the TN quantity refers to the observations where the model successfully avoided assigning the given observation to the label we examine; FP quantity refers to the occasions where the model mistakenly assigned the observation to the class we audit; lastly, the FN quantity refers to the observation that should have been assigned to the label we examine.

**Figure 4:**
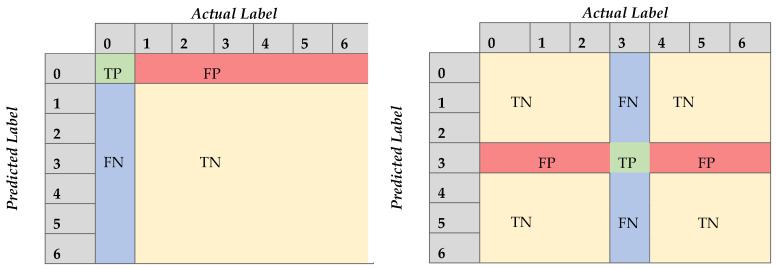
Confusion matrix demonstrations. Left: Class 0 (vessel healthy), right: Class 3 (cylinder); TP: True Positive, TN: True Negative, FP: False Positive, FN: False Negative.

For instance, in our case, a confusion matrix for the needs of our study is defined as a 7 × 7 matrix; to deduce the performance of the model in the multilabel task, we consider each label distinctively, as shown in Figure 4, where indicatively two different labels are considered. Thus, the metric scores of the model in identifying each label are devised, namely, each metric of Table 1 occurs seven times, once for each label, since the notion of the True Positive, True Negative, False Positive, and False Negative changes, as illustrated in Figure 4. 

## 3. Data Collection and Preparation

### 3.1. Data Acquisition

The ship employed in the study is a bulk carrier and its characteristics are listed in Table 2 The initial dataset reflected an operational period of 32 months while the sampling period was set to be 1 min, accumulating approximately 1.5 million measurements. 

**Figure 5:**
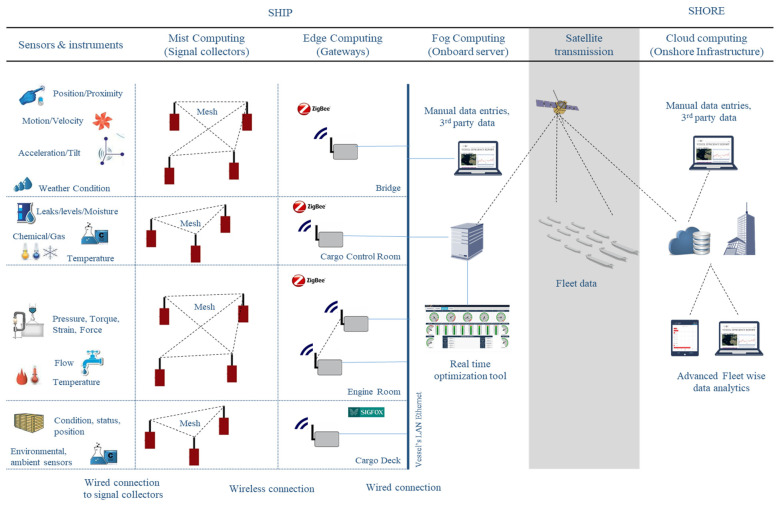
Operational flow of LAROS™ CMS providing synchronized and reliable data.

For the data collection task, the LAROS™ Continuous Monitoring System (L-CMS) was used [31]. The L-CMS allows the synchronized and reliable signals and data collection from any type of sensor, measuring device, instrument, or control system, following the process presented in [32]. Figure 5 presents the overall system architecture. 

**Figure 6:**
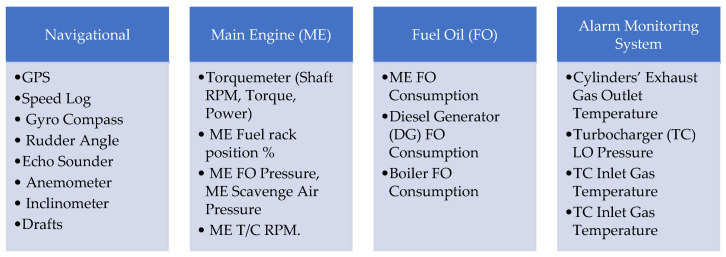
Sources of collected data and the respective features.

More specifically, a secure wireless mesh network (IEEE 802.15.4 MESH) is installed inside each floor of the vessel, based on LAROS™ Smart Collectors. After acquisition, the data are further processed to the gateway periodically and are easily selectable by the end user. Additional layers and payload encryption are used to ensure both quality of service and cyber security at the edge level. This architecture enables the scalability and extendibility of the system together with efficient operations within the vessel’s harsh environment. The preprocessed data are further delivered to the onboard server where they are stored locally for a predefined period (normally 6 months) and are also transferred via normal satellite communication to the next processing level (cloud). At this level, fleetwide data are collected and processed based on dedicated data analytical software tools. Figure 6 presents the signal sources and the specific parameters of interest to our work. With exception of the signals pertinent to the exhaust gas temperature of the main engine’s six cylinders, the purpose of the other parameters was to facilitate the data preprocessing phase of our study, for instance, through the filtering process.

### 3.2. Data Preprocessing

**Figure 7:**
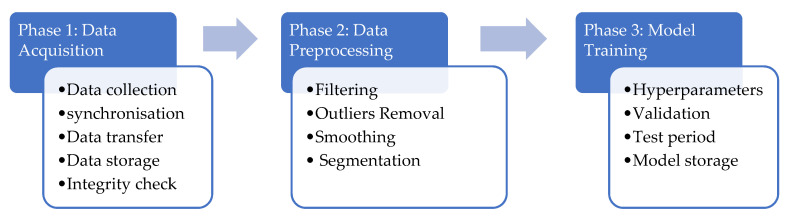
Outline of the study.

As illustrated in Figure 7, the data preprocessing procedure implemented to ensure optimal quality of the data consisted of five steps: (i) filtering, (ii) outlier removal, (iii) data smoothing, and (iv) segmentation. A detailed description of the three first steps is presented in [30]. 

**Figure 8:**
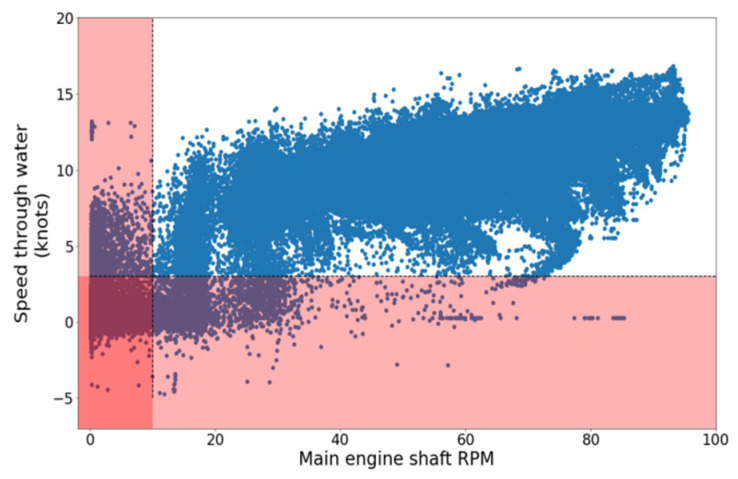
Visualization of filters applied to RPM and ship’s velocity variables.

**Figure 9:**
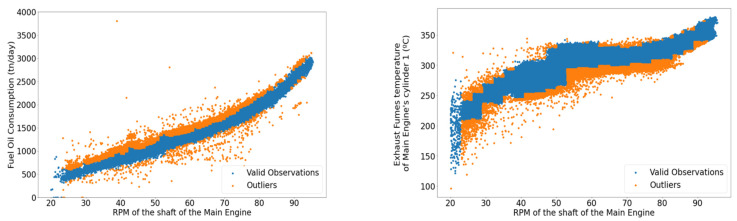
Outlier removal relative to main engine rpm. (**Left**): Fuel Oil Consumption (FOC); (**Right**): exhaust gas temperature of cylinder 1.

**Figure 10:**
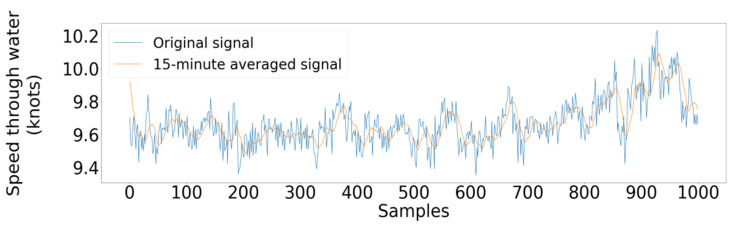
Real (blue line) vs. smoothed (red line) signal obtained with a 15 min averaging window.

According to the procedure described above, two indicative filters are illustrated in Figure 8, with observations lying in the red regions being discarded. Furthermore, in Figure 9, the outlier removal procedure is illustrated, removing extreme values of the fuel oil consumption and exhaust gas temperature at cylinder 1, concerning the main engine’s rpm as a primary parameter, with the samples in orange being discarded, whereas the remainder of the data points appears in blue. Furthermore, regarding the signal smoothing for this study case, it was determined that the 15 min averaging window was adequate, removing most of the noise while also managing to limit the information loss and being capable of following local trends satisfactorily. Figure 10 depicts the smoothed signal as produced by the SMA algorithm contrasted with the original, much more volatile signal.

**Figure 11:**
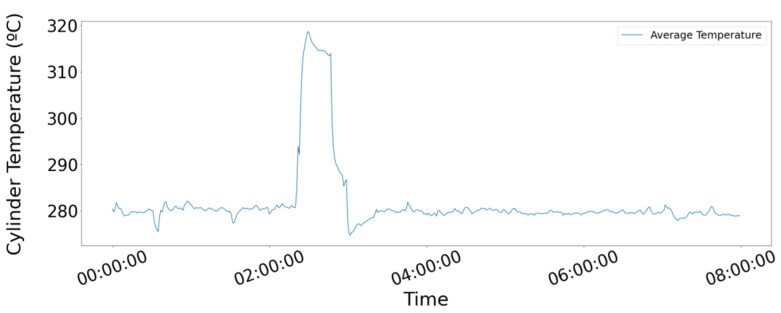
Signal of the average temperature among the main engine’s six cylinders for 8 h.

As a final step, the samples fed into the network must be converted into time series batches. They were generated by segmenting the initial dataset by using a specific time window. In our study, to generate more samples, overlapping between adjacent samples was applied; the overlap percentage was kept at a constant value. The essence of the time window dictates the amount of information depicted by one time series batch, through the period to which it is referring. In our study case, the 1D convolutional model was used to inspect the temperature of the six cylinders of the main engine in an attempt to identify suspicious patterns indicating failure. Indicative time series for the average among the six cylinders are shown over 8 h (Figure 11.). After performing dataset segmentation, in this instance, the finalized dataset array containing the samples could be perceived as a 3D array, whose first dimension refers to the number of available input batches, the second dimension refers to the number of time points that each time series sample contains, and the third dimension equaling the number of features per batch, namely the temperature of the six inspected cylinders. 

Finally, after experimentation, data normalization was found to improve dramatically the performance of our models. The normalization algorithm implemented on this occasion was the Standard Scaler from the Scikit-Learn Library, which is expressed as follows:

(4) $$z=\frac{\left(x-\mu \right)}{s}$$

where *μ* and *s* is the sample mean and the sample standard deviation for the measurements of each standardized variable

## 4. Experimental Results

### 4.1. Application

Convolutional neural networks consist of diverse parameters that profoundly affect the efficiency of the models. Every genre of the deep learning model receives an input, which in our study cases is the input time series, and by properly adjusting internal trainable parameters, such as the neuron weights and biases, generates an output. Nevertheless, training a deep learning model also entails parameters whose values need to be set a priori, called hyperparameters, whose configuration significantly affects the efficacy of the model. Deducing to the optimal hyperparameter configuration is not a straightforward endeavor, requiring experimentation through trial and error. The genre of inspected and tuned hyperparameter along with the audited values, which in essence are the candidate optimal values, are tabulated in Table 3. The multitude of combinations, as can be inferred by the data in Table 3, suggests that finding the optimal configuration with a manual investigation for the best configuration, where various arrangements are randomly selected, is implausible. Therefore, an automated algorithm is required for this endeavor. For that matter, the grid search is selected since it was deemed computationally affordable after experimentation. More specifically, this algorithm exhaustively searches for the optimum across the entire hyperparameter plane.

In our study, a parametric inquiry is presented, aspiring to determine not only whether CNNs are applicable for CBM schemes in marine engineering, but also to examine the effect of significant factors, such as the degree of detected degradation and the input time frame, to the efficacy of the models developed [33]. 

#### 4.1.1. Time Window

**Figure 12:**
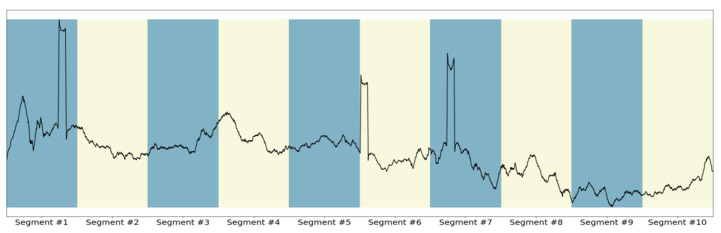
Demonstration of segmentation.

The first inspected parameter is the time window of the input samples. More specifically, the time window defines dataset segmentation into smaller samples reflecting the operational profile over the specific timeframe, dictating the number of samples each input time series batch comprises, along with the final number of available samples to train and assess the model. Indicatively, Figure 12 illustrates the division of a random signal into 10 segments.

**Figure 13:**
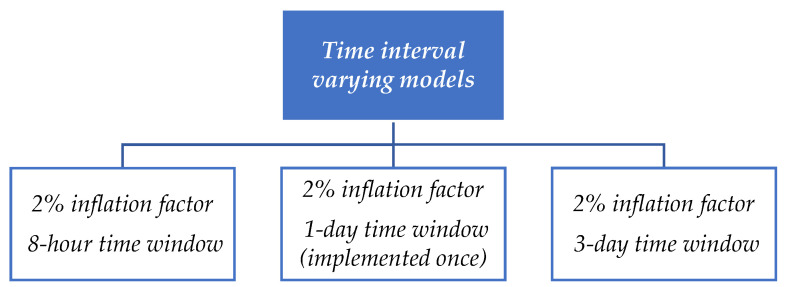
Time interval varying models.

In this regard, it is investigated whether having fewer but wider input time series results in more effective models or vice versa. Figure 13 tabulates the instances with the different inspected time intervals of the input samples, keeping constant the degradation degree induced; information regarding the degradation is presented in the next paragraph. 

First, data segmentation, using an overlap of 90% between adjacent samples, according to the 8 h window results in 10,205 input time series with a length of 475 data points, the 1-day time window leads to the generation of 3930 batches of time series of 1440 data points, and lastly, using the 3-day window results in the creation of 1280 time series samples, each containing 4320 time stamps. It should be noted that the overlap percentage was implemented to generate more samples. 

Moreover, it is very significant to ensure that the dataset contains observations reflecting equally each of the seven possible operational states, so that the dataset is not skewed, thus ensuring that the model is trained equivalently upon all possible conditions. For that reason, the original dataset corresponding to the well-functioning vessel is concatenated with six other similar datasets, with the only difference based in the fact that the artificial damage inducement is undertaken evenly across all the cylinders, ensuring that all seven conditions are equivalently represented in the final dataset. Therefore, comparing with what was explained above, the final number of time series samples generated from each time window was multiplied by seven: the 8 h, 1-day, and 3-day time frames comprised 71,435, 27,510, and 8,960 input time series batches, respectively. 

Lastly, the training, validation, and test subsets are defined. The incentive of the training set consists of the time series dedicated for algorithm learning, hence, requiring the majority of the available samples. The validation set is utilized as means of tracking the progress of the algorithm during training, and the test set consists of unseen samples, to examine the ability of our models to generalize and return the desired results. Applying a 70%–10%–20% percentage distribution in each instance, which is frequently seen in the machine and deep learning community, the samples each of the inspected time windows provide are listed in Table 4.

**Figure 14:**
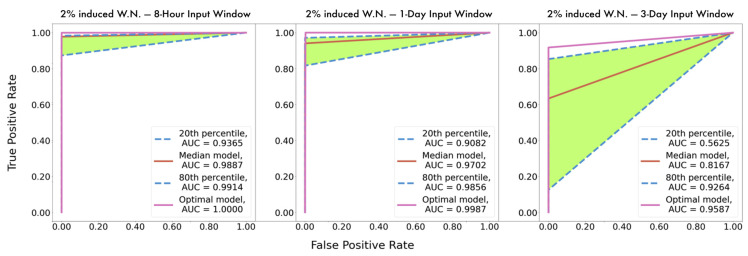
ROC curves and AUC values comparison of corresponding time-varying models.

Having described the dataset composition, the next part of the model development course is the experimentation regarding the optimal configuration of hyperparameters. Considering the entire set of examined models with different configurations of hyperparameters, we ranked them concerning their yielded accuracy. The ROC curves of the models appertained to the 20th, 50th, 80th percentile are plotted along with the curve corresponding to the optimal configuration. This process was executed in all three instances with the different input time windows, as illustrated in Figure 14.

Observation of the following figures evinces most significantly a remarkable performance of the proposed methodology in every instance, as the optimal models on every occasion appear to achieve AUC values proximate to one. This indicates clearly that the proposed methodology achieves with great accuracy identification of failure proneness patterns together with localization of the damage. Additionally, this is further supported by the high metric scores, as shown in Table 5. It should be again emphasized that in a seven-class classification problem, each metric score occurs seven times, for each label, and in Table 6 the averages of these seven values are presented for each metric.

**Figure 15:**
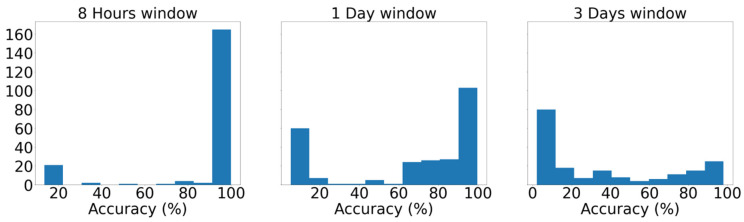
Distribution of accuracy scores of the numerous models during hyperparameter tuning across the three developed instances.

After further observation, it can be discerned that the shrinkage of the input time window results in more accurate instances across the entire spectrum, as comparison among the models appertaining to the same percentiles unequivocally shows that AUC values increase as the window narrows. Moreover, the histograms of Figure 15 illustrate the distribution of the average, among the seven classes, yielded accuracy from the various models at each instance as they occurred during the tuning. Demonstrating that majority of models with the 8 h input window achieve greater than 90% accuracy, whereas the distribution in the instance with the three-day input window shows that most of the models did not present equally remarkable results. This deduction is deemed useful because on the occasion of designing a similar failure detection system, the hyperparameter tuning procedure of the models with the smaller input window would plausibly be considerably less arduous as more configurations achieve remarkable performance. Lastly, as mentioned earlier, Table 5 presents in detail the average scores, among the seven labels, yielded by the optimal model from each instance, verifying the aforementioned findings, as all metrics increase for smaller input windows. 

#### 4.1.2. Induced Degradation

Subsequently, various degrees of induced degradation were also audited, attempting to determine how the efficacy of the proposed networks varied regarding the level of degeneration we aspired to detect. In other words, we examined whether our models could successfully identify even subtle deficiencies contributing to timely recognition of failure proneness. For our study, having available data representing a defective condition of the vessel was essential for training the network. Nevertheless, the data collected through the LAROS™ CMS reflected a well-functioning state of the ship with no signs of degradation. This obstacle was overcome by artificially inducing degradation of the ship by trying to imitate its behavior at the degraded condition intended for detection. More explicitly, white noise was convolved with the original signal of the designated cylinder, which would manifest defective patterns. Regarding the values of the white noise distribution:

- The mean value (μ) of the white noise signal is equal to the respective value of the well-functioning signal, namely the value that it would have if we did not intervene. We denote this as I.S (Initial Signal), the vector of the parameter being altered.
- Its standard deviation (s) is set equal to the multiple of the standard deviation of the healthy signal of the respective feature. We denote this as STD_I.S_ (Standard Deviation of the Initial Signal), the standard deviation of the initial signal.

**Figure 16:**
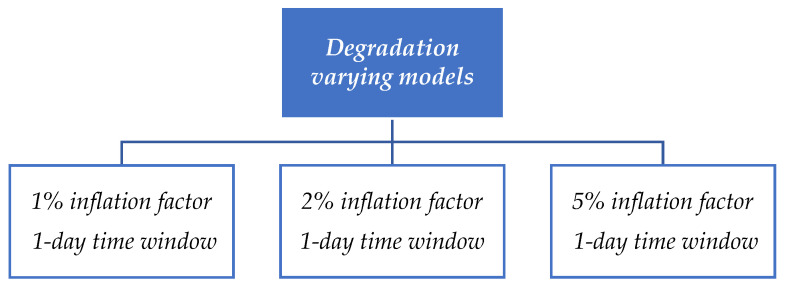
Models developed with diverse levels of degradation and input time horizons.

In other words, the white noise being convolved is governed by a distribution

$$W.N.\;\sim \;N\left(I.S.,{\;k*\mathrm{STD}}_{I.S.}\right),$$

where k is the factor being multiplied with the standard deviation, which causes the inflation of the initial signal, as illustrated in Figure 16. The inspected values of the inflation factor are 1%, 2%, and 5%. Figure 16 shows the instances created with variable jittering factors. 

**Figure 17:**
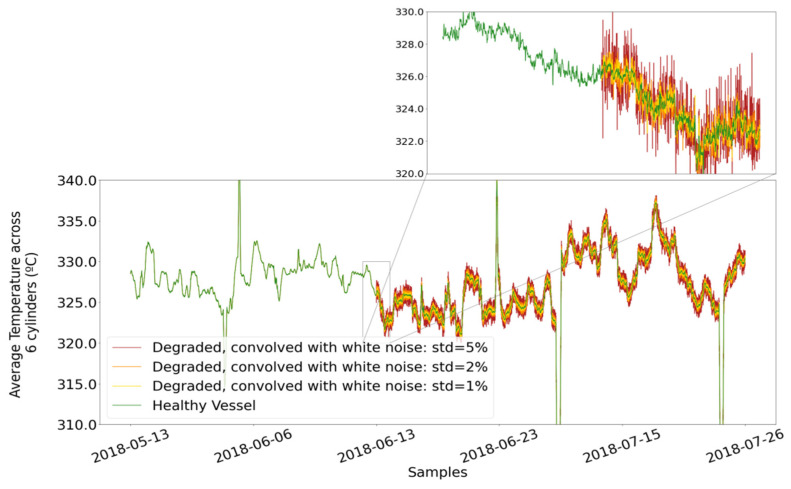
Demonstration of levels of artificially induced degradation.

In Figure 17, the signal of the well-functioning vessel is shown along with the three levels of induced degradation. The yellow-colored signal represents the inflated jitter with the 1% factor and is not easily noticed from the healthy signal with mere observation. The 2% degeneration inducement, shown in darker orange, is slightly easier to distinguish, whereas the signal with the 5% degeneration, shown in red, is significantly more volatile, thus easily noticed.

**Figure 18:**
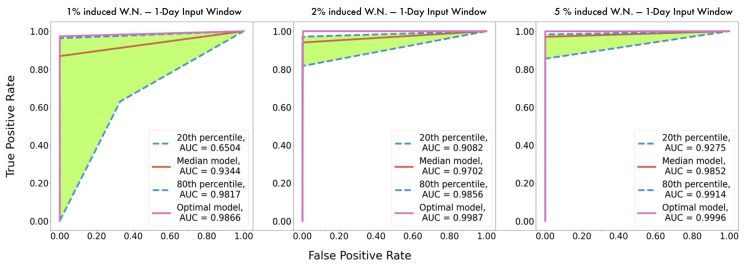
ROC curves and AUC values comparison of corresponding degradation varying models.

**Figure 19:**
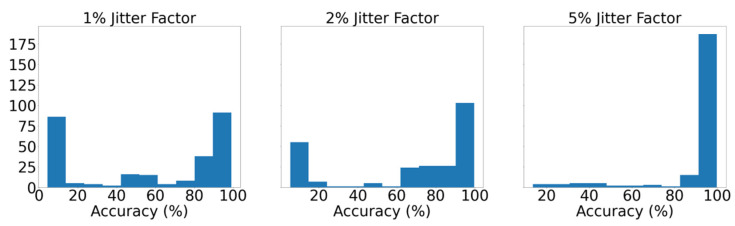
Distribution of accuracy scores of the numerous models during hyperparameter tuning across the three instances developed.

Following the previous pattern, the various models defined by the different hyperparameter configurations, at each instance with the diverse degree of degradation, are ranked according to their efficacy and the ROC curves of the models appertaining to the 20th, 50th, 80th, and 100th (optimal) percentile are plotted, as illustrated in Figure 18. Additionally, the histogram shown in Figure 19 illustrates the distribution of the average accuracy achieved, among the seven classes and across the various audited configurations for the three instances that were developed.

As demonstrated in these figures, the proposed methodology is capable of identifying and localizing adequately all levels of induced degradation that were examined. However, it is also evinced that as the jitter factor increases it becomes more facile for the networks to correctly identify the patterns, which signify defective operational profiles, as more configurations achieve higher accuracy scores. Conversely, the model aspiring to detect the most subtle degree of degeneration requires very careful and thorough tuning to achieve satisfactory accuracy. Lastly, Table 6 tabulates the average metrics, among the seven distinct labels the optimal models across the three instances with the variable degree of artificially induced degradation. The results displayed in this table verify the comments and the conclusions drawn earlier. 

### 4.2. Comparison with Benchmark

After having described the parametric inquiry, how the audited parameters affect the networks and having acquired a better understanding of the behavior of the models along with their remarkable scores on the test set, we proceed to examine and compare the efficiency of the proposed methodology in contrast to well-established machine learning algorithms in the same task and thereupon consider the best performing methodology. For this matter, the ML algorithms introduced and implemented are:

1. Support Vector Machine Classifier (SVMC or SVC)
2. Random Forest Classifier (RFC)

The implementation with the benchmark classifiers was undertaken by extending the inspected instances of the parametric analysis described in Figure 13 and Figure 16, according to the instances shown in Table 7, subsequently developing the respective 1D-CNNs and matching them with two counterpart benchmark classifiers. The dataset utilized is the same as the one used for the implementation of the proposed CNN models, and the percentages of the dataset allocating the input samples to the each subset remain also intact at 70%–10%–20% for training, validation, and test set, accordingly providing uniformity to the comparisons being held. 

**Figure 20:**
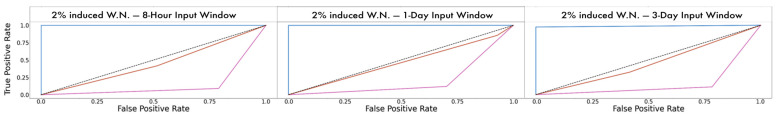
Comparison between proposed methodology 1D-CNN (blue line) and two benchmark classifiers (Random Forest c—pink line, Support Vector—red line) through their yielded AUC values and the respective ROC curves, for the three instances investigated.

ROC curve plots and calculation of the respective AUC are employed to facilitate the comparisons among the different classifiers. First, Table 8 presents the AUC values corresponding to each classifier for every instance. Subsequently, Figure 20 and Table 9 present indicative ROC curves and the respective AUC values, respectively, from selected instances, illustrating the defectiveness of the ML classifiers in the task of the 1D signal classification, in contrast to the near-perfect classification of the proposed 1D-CNNs on every occasion, with AUC scores constantly in the vicinity of 0.99. Additionally, the metric scores of the three classifiers in each of the instances developed and inspected are recorded for verifying the preeminence of the proposed models, achieving near 100% to every metric across all nine instances. Furthermore, on numerous occasions, the accuracy is proximate to 15%, which means that the models cannot evaluate the state of the vessel and randomly predict one out of the seven possible states. 

## 5. Conclusions

In the current work, an effort was made to investigate how deep learning approaches can be employed to exploit a heterogeneous source of information coming from data collected onboard. The model developed was implemented using real case data for the detection and real-time condition monitoring of the primary systems. The work aimed to develop a framework for decision support on maintenance activities in short-, medium-, and long-term time horizons. Furthermore, an in-depth investigation of the effect of multiple variables on the performance of the models was presented. In this context, the parametric investigation was undertaken to attempt to determine how accurately different levels of degradation would be detected, together with the effect of the input time interval on the proposed network’s efficacy. Additionally, due to the absence of data reflecting degraded operational profiles of the vessel under study, artificial damage had to be effectuated, trying to emulate different realistic degradation cases for each methodology to detect. 

Concluding, it was shown that 1D-CNN models can infer the dynamic properties of the vessel components’ decay and status on different time horizons. It was concluded that the short-term FD mechanism benefitted from the shrinkage of the input window, profoundly increasing yielded accuracy in the predictions on the test set. Moreover, regarding the degradation variable models, results indicated that the proposed methodology was more potent as the degradation degree increased. However, it could be claimed that all levels examined were adequately detected, indicating the efficiency of the proposed methodology contributing to the timely failure detection; in contrast, the benchmark ML classifiers presented failed to identify adequately defective patterns for small degenerations. Lastly, it was also evinced that the proposed methodology not only accurately identifies the failure proneness in the system of the main engine, but also manages to localize the occurrence of the trigger event with great accuracy. 

Moving forward, in our future work we will perform a further parametric investigation of the CNN models, to evaluate the efficiency of the results with different damage severity and data sample overlap percentage. Additionally, a parametric study will be investigated, where multiple cylinders are defective in the same instance. Additionally, the next step in this direction will be to assess the efficacy of our models by utilizing real samples that have been pre-established and pre-labeled as defective, since the applicability of the proposed networks has been evinced successfully. Finally, examination of the applicability of the results of a trained model based on the data of a particular vessel to evaluate the condition of another ship of similar type or even considerably dissimilar is amongst our short-term plans. 

## Figures and Tables

**Table 1:** Metrics definition.

Metric Definition	Meaning
$Accuracy=\frac{TP+TN}{TP+FP+TN+FN}$	The ratio of correctly evaluated samples
$Precision=\frac{TP}{TP+FP}$	The rate of correctly labeling a sample as positive
$Recall=\frac{TP}{TP+FN}$	The rate of identifying correctly a positively labeled observation
$Specificity=\frac{TN}{TN+FP}$	The ratio of correctly identifying a negatively labeled observation
$F1=\frac{2\cdot TP}{2\cdot TP+FP+FN}$	The harmonic mean of precision and recall

**Table 2:** Ship characteristics.

Parameter	Value
Length	300.00 (m)
Breadth (Moulded)	55.00 (m)
Depth (Moulded)	25.00 (m)

**Table 3:** Hyperparameter inspected values.

Hyperparameter Name	Values Audited
Number of conv. layers	2, 3, 4
Number of kernel per layer	6, 8, 10, 12, 15
Kernel size	2, 3, 5, 7
Number of Fully Connected (FC) layers	1, 2
Number of nodes per FC layer	3, 5, 7
Learning rate	10^−3^, 7.5·10^−4^, 5·10^−4^, 2.5·10^−4^, 10^−4^, 7.5·10^−5^, 5·10^−5^, 2.5·10^−5^, 10^−5^
Batch size	64, 128, 256, 512,1024
Epochs	50, 100, 150, 200, 300, 400, 500
Dropout rate	0, 0.1, 0.2

**Table 4:** Time series samples.

Input Window	Total Samples	Train Set	Validation Set	Test Set
8 Hours	71,435	50,005	7143	14,287
1 Day	27,510	19,257	27,510	5502
3 Days	8960	6272	896	1792

**Table 5:** Metric scores across the three developed instances with a variable input window.

Model	Accuracy	Precision	Recall	Specificity	F1
8 Hours	100	100	100	100	100
1 Day	99.8	99.9	99.9	100	99.9
3 Days	96.6	96.6	96.9	98.6	96.6

**Table 6:** Metrics scores across the three developed instances with a degradation input window.

Model	Accuracy	Precision	Recall	Specificity	F1
1% jitter factor	100	100	100	100	100
2% jitter factor	99.8	99.9	99.9	100	99.9
5% jitter factor	96.6	96.6	96.9	98.6	96.6

**Table 7:** Extended parametric inquiry for model benchmarking.

	1% Degradation	2% Degradation	5% Degradation
8 hours	1% inflation factor and 8 h, time window	2% inflation factor and 8 h, time window	5% inflation factor and 8 h, time window
1 day	1% inflation factor and 1-day time window	2% inflation factor and 1-day time window	5% inflation factor and 1-day time window
3 days	1% inflation factor and 3-day time window	2% inflation factor and 3-day time window	5% inflation factor and 3-day time window

**Table 8:** AUC values of each classifier in each inspected instance.

	8 Hours			1 Day			3 Days		
% Induced Damage	1%	2%	5%	1%	2%	5%	1%	2%	5%
Proposed	0.998	1.0	1.0	0.995	0.997	1.0	0.945	0.987	0.991
RFC	0.194	0.271	0.440	0.155	0.208	0.510	0.092	0.164	0.381
SVC	0.459	0.495	0.548	0.460	0.464	0.471	0.436	0.453	0.467

**Table 9:** Metrics scores of the three classifiers in three indicative instances.

	Accuracy			Precision			Recall			Specificity			F1		
Model *	1	2	3	1	2	3	1	2	3	1	2	3	1	2	3
Proposed	99.9	99.3	99.8	100	99.6	99.9	99.9	99.6	99.8	100	99.7	99.9	99.8	99.6	99.8
RFC	18.3	15.2	23.6	13.8	10.8	17.9	12.9	9.4	15.3	29.1	21.7	31.4	15.6	10.0	16.3
SVC	46.2	46.0	45.9	44.7	43.8	46.2	51.5	25.6	69.6	33.7	66.8	23.6	47.1	32.3	55.6

* 1: 1% inflation factor and 8 h time window. 2: 1% inflation factor and 1-day time window. 3: 2% inflation factor, and 1-day time window.

## Data Availability

Not applicable.

## Appendix A. Comprehension of CNN Information Flow

The following Table presents a comprehensive overview of the operations taking place in a 1D-CNN.

**Table A1:** Metrics scores of the 3 classifiers in 3 indicative instances.

Name	Symbols/ Equation	Notion
Input to the networks	1D tensor	Time series as 1D input tensors *X* consisted of *mXn* pixels
Convolution and Activation function	Z(x)=ReLU(K°S(x))	Kernel sliding through the input tensor received by each layer. The activation function introduces nonlinearity.
Max Pooling Layer	G(x,y)=max(P°S(x,y))	Dimensionality reduction decreasing the computation cost
Multilayer Perceptron and Activation function	$z^{\left[l\right]}=ReLU\left(W^{T}\cdot a^{\left[l-1\right]}+b\right)$	The 2D tensor from the last convolution or pooling layer is flattened and inserted into an MLP to carry out the classification task.
Classification	$a^{\left[L\right]}=Softmax\left(W^{T}\cdot a^{\left[L-1\right]}+b\right)$ $=\frac{e^{z_{i}^{\left[L\right]}}}{\sum_{i=1}^{M}e^{z_{i}^{\left[L\right]}}}$	The output of the last L^th^ layer.
Loss function	$L=CCE(y,\;\hat{y}$) $=–\overset{M}{\underset{i=1}{\sum }}y_{i}\log a^{\left[L\right]})$	The main objective of the networks’ optimization concerns the minimization of the loss function.
Derivation	$\frac{\partial L}{\partial w_{i}}$ & $\frac{\partial L}{\partial b_{i}}$	The model learns through back-propagation. Only learnable parameters in a 1D-CNN are the weights and biases from both convolution layers and MLP.
Self-Optimization	$w_{i}=w_{i}-a*\frac{\partial L}{\partial w_{i}}\;b_{i}=b_{i}-a*\frac{\partial L}{\partial b_{i}}$	Updating the weights and biases as shown. The term α denotes the learning rate of the optimizer, signifying how quickly the algorithm converges.
